# The Greatest Thing in the World

**Published:** 1907-02

**Authors:** R. E. Ware

**Affiliations:** Shelby, N. C.


### THE GREATEST THING IN THE WORLD

By R. E. Ware, D. D. S., Shelby, N. C.

What a great and sublime thought was that conceived in the mind of God when He determined to create the world.

To measure and weigh the matter and force necessary to form the globe, to determine the elements required to do the work lying through the years before it, to assign to them their laws, specify gravities and affinities beforehand together with all the combinations and collocations they were capable of making required the mind and hand of an omnipotent and omnicient God.

Had creation stopped here, what a barren and useless world this would have been! But it was not the purpose of the Creator to do this. All sea and land and air were filled with living, moving things and each in and adapted to its proper element. The earth must turn on its axis, dividing day and night and revolve round the sun in regular order that the seasons may come and go and all proper conditions brought about to sustain life. That there was a great purpose present from the beginning, directing and controlling, there can be no doubt. "It presided over the first nebulous mist that floated out to take form in the foundations of the earth, but the meaning of creation was not understood till dust stood erect in a living man."

If it required a great thought to create a world, a much greater was necessary to create man. In all creation two essentional things are present, mind and matter. That mind is superior to matter there is no doubt. Created in the image of God, receiving the breath of life and thus made partaker of the divine nature, we see in man the final expression of the creative process and he at once becomes the interpreter and interpretation of all that had gone before. Superior to all created beings, he was given power over all things on the earth, but not until "the ocean beds were dug out and the waters called off from a part of the earth's surface; the mighty peaks and the majestic turrets of the mountain chains were lifted into the sky; the poisonous forces destructive of man's life locked up in soils and rocks; the meadows sown with grasses and the hospitable arms of the trees loaded with fruit, then upon the earth, adorned and ready for his coming, man appeared.'' Placed in the garden of Eden, he looked out upon this great, grand, beautiful, good world, created for him and over which he was placed as ruler. Little knowledge had he of its vast and varied resources and possibilities. The air was filled with birds, the sea with fish, beast and creeping things roamed over hill and plain. Before all this Adam stood amazed and confused. The hum of factory and mill was not yet heard, no thunder of the locomotive sounded through the valleys, no steamboat crossed rivers and seas, neither had electricity been harnessed and subjected to the human will.

Six thousand years have elapsed since creation. Had our first parents remained in the garden of Eden, perhaps the multiplied thousands of the discoveries and inventions of modern civilization would never have been known. Adam's knowledge, as yet, had all come by divine revelation and not by necessity or experience. Had he remained in the garden, perhaps he might always have been a kind of an artificial, kindergarten man. But God did not so intend it. After viewing in awe-struck silence all the wonderful glories and beauties of the world, Adam lay down upon the verdant banks of one of the most beautiful lakes in the garden, filled with wonder, love and praise, but weary and lonely he fell into a deep sleep. When he awoke he beheld near his side the most beautiful being in the world—woman—the helpmeet and companion of man. But alas! in an evil hour sin entered the world, and with his brazen and impudent face, flattering and seductive tongue approached the woman. Yielding to his tempting words she discovered God, caused Adam to sin, and they were driven from the garden. Thorns and briars sprang up out of the ground and Adam was told ''by the sweat of his face should he eat bread all the days of his life.'' Though God was displeased with him, yet He commanded him to go forth and subdue the world, increase and multiply and replenish the earth. But all this history, together with the long, varied, severe, but systematic training that followed is familiar and interesting to us all. Out of the family grew the tribes, from the tribes the nation, from the nation the kingdoms, empires and republics.

"By lifting one corner of the veil that hides from us the primitive conditions of mankind, we find that human relationship or society had its origin in the human family. In the care of childhood, where several children were successively born to the same parents, the ties of kinship became firmly knit, sympathies developed and selfishness was dethroned." With primitive man, food was the prime necessity of life, and as long as it was obtainable only by hunting, fishing, or otherwise seizing upon edible objects, fighting, quarreling and everlasting slaughters were inevitable and always resulted in the survival of the fiercest fighters to perpetuate their kind. It was soon noticed that the most coherent families prevailed over their less coherent rivals. The strong ties of family life supplied the motives of peaceful co-operation, which has gradually progressed through the centuries and become more noticeable during the last few decades.

As civilization advanced and population increased, man became more powerful and intelligent. It was discovered that food could be obtained otherwise than by hunting and fishing. Agriculture became an active industry. Commercial relations were established and this permitted a vastly greater population to live on a given area, thus favoring in many ways social organization. The great influence of organized social force is unlimited. "Commerce insures the union, and establishes the relations that make this force possible. It brings men together in the exchange of food, clothing and other necessaries of life, thus enabling them to find their intellects and what thew know, their hearts and what they can love, their wills and what they can do."

The satisfaction of hunger, the accumulation of wealth, and the establishment of social relations were not the only good accomplished, but the greatest benefit was the development of man. In some notable instances this development was neglected. For example look at Babylon. In splendor and wealth, she was without a rival among ancient nations. Situated in the most fertile portion of the globe, her capital eclipsed all others in magnificence. Her hanging gardens were the wonder of the world, but she put no estimate upon her men, except in the amount of labor performed. Luxury, sensuality, drunkenness, utter stupidity and depravity marked the ruin and overthrow of her proud empire, and she had not a man mentally endowed to write her history. What a contrast in the two centuries between 500 and 300 B. C., was that of Greece, who deposited her life in her great men! Her temples have fallen, her Parthenon is in ruins, and her art treasuries are no more, but her history and philosophy are immortal because she emphasized men more than the things they created. But when Greece counted life cheaper than art she no longer had men to create new art and none to protect the old. "By removing the emphasis from men to things, she descended from the Crœsus to the pauper of civilization." Israel had her Moses greater than the tabernacle, David greater than his harp, Isaiah greater than his song, but when she emphasized the forms of worship more than the spiritual, her devotion died and her manhood was destroyed. The same might be said of Rome and other ancient nations. When Rome valued her men more than the laws they formulated, she sat upon her seven hills, the crowned mistress of the world, but when she stressed her laws to the obliteration of her men, she weakened and fell.

Leaving ancient scenes for awhile, let us consider some of modern times. From the time the first auroral beams of the sun kisses the golden sands of our eastern shores till the shining rays are lost behind the western slopes, he looks down upon the greatest country and the greatest people in the world.

The United States is the home of science, discoveries and inventions, the birthplace of orators, warriors and statesmen, but in the fierce competition existing and growing in the commermercial world today, we see the attempt to re-enact in business life "the survival of the fittest in the struggle for existence." This spirit belongs to the dark ages, to beasts of the jungle, and should be forever relegated to its place. In the many manufacturing establishments we see men who are estimated more for what they can make than for the spirit or social nature that is within them. Boys and girls who ought to be in school are denied the privilege because they are worth 40 cents a day in a factory. We are using too many of our people as a commodity. There are a great many mowers, reapers, engines, cotton gins, hats, shoes. pins, buttons, countless numbers of fabrics, tools and machines of different kinds turned out from our mills and factories every day. If, in the making of these things, every person employed does not make a man out of himself, the whole thing is a disastrous failure. Our beautiful Southland is fast becoming a manufacturing center. Let us see to it that our manhood is not lost in the product of machinery. In this strenuous age, in our hurry to get rich quick, we too often allow our boys and girls to rush into business and neglect the culture of their minds. This should not be so, for we find in man a threefold nature—body, mind and spirit. These must be harmoniously developed if he is to be the interpreter and interpretation of the physical universe. "Never was the thought of man absent from her movements through Pliocene, Miocene, Eocene, Cretacious, Jurassic, Triassic, Carboniferous, Devonion, Silurian or Cambrian ages." When the morning stars sang together the thought of this grand masterpiece of creation gave melody to their song.

But what is it that makes man "The Greatest Thing in the World"? Is it the wonderful mechanism of his body, over which physiologists and anatomists have stood confused? No. It is that immortal, divine, God-given nature imparted to him by the Creator in the beginning. By willing, directing and controlling the hands of men, the mind has been enabled to construct the grand fabric of modern civilization. How pleasing and instructive to contemplate the miraculous possibilities of the human mind! Created to interpret nature, it reads her history in the rocks, coal beds, sands, shells and fossils of the primal ages. It discovers the laws of chemistry written in the shape, sizes, affinities and specific gravities of the atoms that enter into the composition of all natural bodies. By observing the movements of the heavenly bodies, the mind sees books on astronomy written in their orbits. "The first books on zoology were written in the structure and habits of the lower animals." The books that fill all libraries says one, are but transcripts from the original volumes written in rocks, seas, flowers, and skies, and as Dr. Martineau has well said, "Man is the only being who can read and transcribe these wonderful volumes." To the ignorant and uneducated mind they remain sealed and hidden forever. To the cattle and beasts of the fields, grass and herbs mean nothing but food; to man, however, every blade and every leaf has a message poetic and beautiful. "To him, who in the love of nature holds communion with her visible forms, she speaks a various language." He hears the song of the muse in the babbling brook, catches the secret of the seasons in the whisper of the winds and attends the warnings of God in the thunder storm.

By research and experience we find that the mind has two eyes, an eye of sense and an eye of reason. The one looks out upon a world of matter and fact, and the other beholds a world of idea and relation. Through the eye of sense the flash of the lightning is seen in the cloud, through the eye of reason it is harnessed and subjected to the human will. The eye of sense observes the steam lift the lid of the kettle, the eye of reason sees the locomotives and steamships with bands of steam and steel linking the continents together, laying the products of every nation at our door, and placing every race on the globe face to face. The eye of sense may observe the rough marble slab a thousand times, but the eye of reason looks beneath the surface and beholds the face of an angel. Through the eye of reason ideas of beauty are turned into painting, and Raphael's "Transfiguration" blesses the world. "Ideas of harmony are turned into song, and Handel's 'Messiah' agitates the thoughts and feelings of men with the melody of the skies. Ideas of form are turned into sculpture and Michael Angelo's 'Moses' augments the world's fund of conviction and courage." By the proper manipulation and adjustment of mine, forest and canebrake, Beethoven, Handel and Mozart, by their genius were enabled to bring out the matchless melodies and sacred symphonies God had put into them in the beginning. Notes of some song are in every created thing. They only need the hands of a master to find and play upon their mystic keys. The mind, itself, is a many stringed instrument, and when played on by the hand of divinity, can charm the universe into submission.

Astronomers tell us that the earth is very small in comparison to countless other inhabited worlds. This may be true or not, but we do know that this is the greatest world of which we have any certain knowledge, that man is the greatest being in the world, and that our Creator is a great God.

Seeing then that man is the greatest being in the greatest world, endowed with mind and spirit, intellect and reason, whose capabilities are without limit, whose possibilities are boundless; susceptible to the highest culture, of the purest motives, of the noblest thoughts and loftiest aspirations; and that he may also become debauched and dethroned even of reason, and dragged to the lowest depths of degredation and ruin by yielding to the alluring temptations of Satan; and believing by the revelation of the Bible that the spirit is immortal; the harmonious development of the body, mind and spirit is readily admitted to be of supreme importance. Neglect this and man is not complete. The divine image will not be perfectly reflected in him. Let it be despised and a great chasm is left unspanned in this life and a still greater one in the life to come. Shall we not, therefore, educate in unison the body, mind and spirit, purify and refine them together? Then we shall feel assured that when we approach our final sleep, "The stars shall fade away, the sun, himself, grow dim with age, and nature sink in years; but we shall flourish in immortal youth, unhurt amid the war of elements, the wreck of matter and the crash of worlds."

